# Editorial: Diversity, ecology and evolution of archaeal viruses

**DOI:** 10.3389/fmicb.2023.1333790

**Published:** 2023-11-20

**Authors:** Lu Fan, Junfeng Liu, Christian Rinke, Brett J. Baker, Changyi Zhang

**Affiliations:** ^1^Shenzhen Key Laboratory of Marine Archaea Geo-Omics, Department of Ocean Science and Engineering, Southern University of Science and Technology (SUSTech), Shenzhen, China; ^2^Institut Pasteur, Paris, France; ^3^Australian Centre for Ecogenomics, School of Chemistry and Molecular Biosciences, The University of Queensland, Brisbane, QLD, Australia; ^4^Department of Marine Science, University of Texas at Austin, Marine Science Institute, Port Aransas, TX, United States; ^5^Department of Integrative Biology, University of Texas at Austin, Austin, TX, United States; ^6^Carl R. Woese Institute for Genomic Biology, University of Illinois at Urbana-Champaign, Urbana, IL, United States

**Keywords:** archaeal viruses, ecology, evolution, diversity, genetic tools

A prominent feature of archaea is the remarkable diversity of their viruses. Archaeal viruses comprise a vastly unexplored part of the global virosphere. Many archaeal viruses do not share evolutionary relationships, structural features, or genomic characteristics with viruses of bacteria or eukaryotes. A broader diversity in morphology of archaeal viruses, compared to bacteriophages, suggests a long and complex evolutionary history. Recent cultivation and metagenomic-based studies have uncovered several novel groups of viruses that infect archaeal extremophiles and mesophiles in diverse habitats. Viral ecological research has revealed important roles of archaeal viruses in influencing microbial communities, in particular in aquatic ecosystems. Many archaeal viruses are also promising candidates for genetic tool development due to their stability in extreme physiochemical conditions.

In spite of several pioneer studies, the vast diversity of archaeal viruses and the interactions with their hosts still remain explored. Isolating archaeal virions from their natural environments is obstructed by the difficulty in culturing their archaeal hosts. Metagenome sequencing has revealed a tremendous amount of potential viral sequences from environments around the globe. A considerable proportion of them likely belong to archaeal viruses. However, few of these sequences have been classified because of the limited reference databases of archaeal viruses. The goal of this Research Topic is to promote publications of recent advances in archaeal virus research. Three original research and one review article published under this topic described the features of both archaeal viruses and their archaeal hosts isolated from extreme environments, as well as the vast diversity of uncultured marine archaeal viruses in global brackish waters.

Viruses that infect crenarchaea, especially those within the order *Sulfolobales*, are among the earliest studied archaeal viruses yet their structural and functional diversity in natural hydrothermal environments is still largely unexplored. Overton et al. reviewed the STIV-like viruses in the family *Turriviridae* infecting *Sulfolobus* species. They detailed the virion structure including the capsid, the turret-like spikes, the internal lipid envelope, the glycosylation of the major coat protein, and the packaging of genome. The host-lysis mechanisms including the viral attachment to the host and the viral hijacking of the host cell division machinery were further discussed, along with the cultivation and genetic operation systems of these viruses.

Feng et al. isolated and characterized five novel viruses of the thermophilic archaeal family *Sulfolobaceae* from hot springs in Yunnan, China. The diverse protein capsids of these viruses were either icosahedral, filamentous, or spindle-shaped and the lipid envelopes were either inside or outside of the protein capsids. Specifically, the lipid species of these enveloped viruses and their archaeal hosts were identified, demonstrating virus-host linkage in lipid composition. These viruses are candidates for the development of novel virus-host models for genetic studies of archaeal viruses.

Tittes et al. developed new virus-archaea operational models, by functionally characterizing the archaeal host of *Haloferax* tailed virus 1 (HFTV1)—*Haloferax gibbonsii*. LR25. Genome sequencing analysis showed that this virus lacked a CRISPR-Cas system, which may explain its susceptibility in viral infection.

Lastly, Xu et al. studied the diversity of magroviruses, the potential viruses infecting marine group II euryarchaea (*Ca*. Poseidoniales), in brackish environments. The analysis of genome sequences in global estuaries and enclosed seas uncovered the vast diversity of this novel viral group. Magroviruses were present abundantly both in brackish and open ocean samples with some showing habitat specification and others having broad spectrums of distribution between different habitats. Genome annotation suggested they may be involved in regulating multiple metabolic pathways of MGII archaea.

In conclusion, there is much to be learned about archaeal viruses, including the accurate estimation of their overall diversity, their multiple origins and evolution, their typical or atypical virion structures or life cycles, novel defense systems of their archaeal hosts, the mechanisms of archaea-virus interactions, and their ecological contributions. The further development of molecular tools based on the cultivation and characterization of novel archaea and their viruses are needed to address these gaps in our knowledge. Moreover, technologies to identify viruses infecting archaea directly from environment, visualization methods to assess virion structure, and innovative bioinformatic tools are required to elucidate the mysterious origins and evolution of archaeal viruses.

## Author contributions

LF: Writing – original draft, Writing – review & editing. JL: Writing – review & editing. CR: Writing – review & editing. BB: Writing – review & editing. CZ: Writing – review & editing.

